# Effect of Ion Selectivity on Current Production in Sewage Microbial Fuel Cell Separators

**DOI:** 10.3390/membranes12020183

**Published:** 2022-02-03

**Authors:** Ryoya Itoshiro, Naoko Yoshida, Toshiyuki Yagi, Yuriko Kakihana, Mitsuru Higa

**Affiliations:** 1 Department of Civil Engineering, Nagoya Institute of Technology (Nitech), Nagoya 466-8555, Japan; r.itoshiro.994@stn.nitech.ac.jp (R.I.); yagi.toshiyuki@nitech.ac.jp (T.Y.); 2 Graduate School of Sciences and Technology for Innovation, Yamaguchi University, Yoshida, Yamaguchi 753-8511, Japan; kakihana@yamaguchi-u.ac.jp (Y.K.); mhiga@yamaguchi-u.ac.jp (M.H.)

**Keywords:** microbial fuel cell, wastewater treatment, anion exchange membrane, cation exchange membrane, membrane resistance

## Abstract

This study compared the performance of two microbial fuel cells (MFCs) equipped with separators of anion or cation exchange membranes (AEMs or CEMs) for sewage wastewater treatment. Under chemostat feeding of sewage wastewater (hydraulic retention time of approximately 7 h and polarization via an external resistance of 1 Ω), the MFCs with AEM (MFC_AEM_) generated a maximum current that was 4–5 times greater than that generated by the MFC with CEM (MFC_CEM_). The high current in the MFC_AEM_ was attributed to the approximately neutral pH of its cathode, in contrast to the extremely high pH of the MFC_CEM_ cathode. Due to the elimination of the pH imbalance, the cathode resistance for the MFC_AEM_ (13–19 Ω·m^2^) was lower than that for the MFC_CEM_ (41–44 Ω·m^2^). The membrane resistance measured as the Cl^−^ mobility of AEMs for the MFC_AEM_ operated for 35, 583, and 768 days showed an increase with operation time and depth, and this increase contributed minimally to the cathode resistance of the MFC_AEM_. These results indicate the advantage of the AEM over the CEM for air-cathode MFCs. The membrane resistance may increase when the AEM is applied in large-scale MFCs on a meter scale for extended periods.

## 1. Introduction

Wastewater treatment is becoming extremely important in present times. It is crucial not only in conserving public water quality but also in supplying sufficient quantities of safe water to address rapidly increasing demands [1]. Water treatment technology is widely used. However, electricity consumption in sewage treatment accounts for 0.7–4% of domestic electricity consumption in developed countries [2,3], and a substantial amount of greenhouse gases are emitted. To ensure sustainable water cycles, the wastewater treatment should maximize energy conservation by recovering the biomass energy contained in wastewater [4]. Typically, most of the energy recovered is obtained from converting sludge biomass into biogas [4] via microbial fermentation and solid fuel [5]. However, because of the low biomass concentration and substantial volume of wastewater, energy recovery from wastewater is still in the research stage. Several treatment methods that integrate energy recovery, such as microalgae cultivation, membrane-enriched fermentation, and microbial fuel cells (MFCs) [6], have been evaluated in the past. Among these, MFCs received the most attention for treating wastewater with simultaneous recovery of electricity [7].

MFCs generally comprise an anode, a cathode, and a separator. The anode collects electrons that are emitted by the microbial oxidization of organic matter in an anolyte, such as wastewater. The cathode reduces oxidants such as oxygen using electrons collected in the anode. The separator insulates the electrodes while maintaining the mobility of the ions or oxidants. Air-cathode MFCs are the most common type of MFCs and have a single anolyte chamber with cathodes exposed to the air. The catalysts for the anode reactions are microorganisms that degrade various organic matter, and the catalysts for cathode reactions are carbon particles that can reduce oxygen. Currently, the limited electric power production of the MFCs makes them impractical for application in real wastewater treatment. The performance of all three key components needs to be improved [8], particularly in terms of scaling-up. The challenges include developing (i) low-cost mass production technology for the anode, (ii) improved oxygen reducing catalyst for the cathode, and (iii) a resilient separator with high ion mobility. Among them, the selection of the separator significantly affects the performance of the MFC and the lifetime required to generate electric power.

The first air-cathode MFCs were equipped with proton-exchange [9] and later equipped with ion-exchange membranes (IEMs) because of cost effectiveness. Both membranes have been extensively used in hydrogen fuel cells [10] and iron-based flow-battery systems [11]. Non-ion-exchange membranes (NIEs) have been used as separators [12,13,14,15] in combination with polytetrafluoroethylene PTFE) gas diffusion layers (GDLs), which have oxygen mobility [16]. Examples of such membranes include glass fiber mats, polypropylene porous plastic plates, and non-woven fabrics. Compared to IEMs, systems with GDL and NIEs enable lower costs, although the cathode facing the liquid phase generally has a short lifetime due to biofouling on the cathode [17]. The MFCs with GDLs and NIEs exhibited a 34% decrease in electricity production after one month [13]; washing was required to recover the initial levels of performance [18,19] that was caused by aerobic bacterial growth on the cathode facing the wastewater. In contrast, the MFCs equipped with a cation exchange membrane (CEM) performed almost consistently over an entire year [20,21]. Recently, a scalable air-cathode MFCs equipped with an anion exchange membrane (AEM) was demonstrated for the first time [2], and electricity was successfully recovered from sewage wastewater over a year [22]. A comparison of electric power production by MFCs using AEMs and CEMs demonstrated the advantage of AEMs, which mitigate the pH imbalances that are often observed in MFCs with CEMs [23,24,25]. However, comparative studies are limited to milliliter scales in batch mode, making it difficult to evaluate the effects on the performance of MFCs in actual wastewater treatment.

In this study, the effect of the ion selectivity of IEMs on MFCs performance was evaluated in air-cathode MFCs equipped with AEMs and CEMs. These MFCs were operated in the chemostat in a sewage wastewater treatment plant, and the electric power density, chemical oxygen demand (COD) removal efficiency (COD-RE), membrane resistance, and cathode resistance were compared. In addition, the effect of operation time on the AEM resistance was evaluated to estimate the lifetime of the AEMs in the air cathode-MFC.

## 2. Materials and Methods

### 2.1. MFC Used in the Experiment

In this study, a cylindrical MFC core (Φ5 cm × 100 cm) with an air cathode [26] prepared from a stainless-steel mesh surrounded by a carbon-based cathode was used along with an IEMs and nonwoven graphite fabric (TOYOBO Co., Ltd., Osaka, Japan). The IEMs were either CEMs (CSE; Astom Co., Ltd., Tokyo, Japan) or AEMs (ASE; Astom Co., Ltd.). The thicknesses of the CEM and AEM were 0.16 and 0.15 mm, respectively. The membrane resistances were 0.18 and 0.26 Ω·m^2^, respectively, in 0.5 N NaCl at 25 °C according to the manufacturer’s catalogue. Carbon cloths painted with a mixture of activated carbon and carbon black were used as cathodes. Twelve carbon brushes (Φ4.0 cm × 100 cm) were placed around the MFC core, in addition to the nonwoven graphite fabric. The MFC units of the core and 12 additional carbon brushes were defined as the MFC_AEM_ and MFC_CEM_ equipped with an AEM and a CEM as separators, respectively. A mixture of poly (diallyldimethylammonium chloride) (PDDMAC) and PTFE [2] were used as the binders of the carbon catalyst pastes for the MFC_AEM_ and a 7 L/mg-mixture of carbon in a 10% Nafion solution were used for the MFC_CEM_, respectively. Carbon brushes were manufactured using carbon fabrics (T300B-3k-40B, Toray, Tokyo, Japan), soaked in acetone, and heated at 450 °C for 5 h before use [27] to render the surface sufficiently hydrophilic for microbial adhesion [28].

### 2.2. Operation of the MFC Reactors

The MFC_AEM_ and MFC_CEM_ were installed in cylindrical reactors (Φ25 cm × 110 cm) made of polyvinyl chloride and filled with 50 L of wastewater. The reactor was operated in a sewage wastewater treatment plant (Nagoya City) and continuously supplied with influent using a tubing pump (TP-20SA, AS ONE, Osaka, Japan) with a hydraulic retention time (HRT) of 2.6–9.8 h. The HRTs were set by changing the inflow rate to realize different COD concentrations in the reactor. For the initial 35 days, the influent of the primary sedimentation tank (PST) was used; later the effluent of the PST was used due to the clogging of the tube by the influent. Wastewater in the reactor was circulated at a circulation time of 15 min using a submersible pump (LEDGLE, Shenzhen, China). The cathode and anode of the MFC were connected via an external resistor (R_ext_) of 1 Ω and a voltage data logger (VR-71; T&D Co., Nagano, Japan) parallel to R_ext_. The cell voltage between anode and cathode was recorded hourly.

### 2.3. Power Density Curve

Power density curves were measured for the MFC_AEM_ and MFC_CEM_ with HRTs of 3.0–9.8 h at 9, 18, and 30 days after the commencement of operation. As noted previously, the MFCs were connected in parallel with 1, 2, 5, 10, 20, 50, 100, and 10,000 Ω R_ext_ [29]. An Ag/AgCl reference electrode (RE-1B; BAS Co., Ltd., Tokyo, Japan) was installed close to the MFC anode, and the anode and cathode potentials were measured. The anode (R_an_) and cathode (R_ca_) resistances of the MFC were calculated by dividing the electrode potential difference by the current and multiplying it with the separator area [30].

### 2.4. COD Removal and Coulombic Efficiencies

COD was determined by adding potassium dichromate as an oxidizing agent and mercury as a reducing agent to the sample, followed by heating. Subsequently, the absorbance was measured with a spectrophotometer to determine the concentration [31]. The *COD-RE* [%] was calculated using Equation (1) with *COD_IN_* [mg/L] and *COD_EF_* [mg/L] values, that is, the concentrations of organic matter in the influent and effluent, respectively.

(1) $$COD-RE=\frac{COD_{IN}-COD_{EF}}{COD_{IN}}\times 100.$$

Coulombic efficiency (CE) is the ratio of charge recovered as current to the total charge obtained from the decomposition of organic matter in sewage. It can be expressed by Equation (2).

(2) $$CE=\frac{C_{p}}{C_{Ti}}\times 100.$$

where *C_p_* [C] is the cumulative charge carried by the current during the given HRT and *C_T_* [C] is the theoretical charge that is calculated using Equation (3).

(3) $$C_{T}=\frac{∆COD\cdot VFb}{M}\;.$$

where *ΔC* [g/L] is the difference between *COD_IN_* and *COD_EF_*, *V* [L] is the volume of wastewater in the reactor, *F* [C/mol] is Faraday constant (=96,485 C/mol), *b* is number of moles of electrons produced from 1 mol of oxygen (*b* = 4), and *M* [g/mol] is molecular weight of oxygen (*M* = 32). The *CE* was determined by least-squares fitting of the measured and calculated currents using the *CE* as a variable in Equation (2).

### 2.5. Measuring Membrane Resistance

The electrical resistance of the AEM before and after 35, 583, and 768 days of operation was measured using a custom-built acrylic cell, as described in a previous study [32]. The old AEMs (583 and 783 d) were taken from an air-cathode MFC [22] similar to the MFC used in this study. The AEMs were taken from the top, middle, and bottom of the cell, and cut into squares of approximately 5 cm × 5 cm (with an effective area of 11 cm^2^). Following this, the cell was filled with a 0.5 M NaCl solution. Two parallel Pt electrodes were placed on both sides of the AEM and connected to an LCR meter (AD-5827; A&D Co., Ltd., Tokyo, Japan). The membrane resistance (R_m_) [Ω·cm^2^] was calculated using the values of resistance measured between the electrodes with and without membranes, denoted by R_1_ and R_0_, respectively, using the equation R_m_ = R_1_−R_0_. The resistances were measured using alternating current (AC) at a frequency of 10 kHz. It must also be noted that certain membranes were pre-incubated in 0.5 M NaCl.

### 2.6. Linear Sweep Voltammetry (LSV) Test

The effect of the AEM ages on the cathode reaction was evaluated by monitoring the current in a small cylindrical reactor (Φ6 cm × 5 cm) equipped with 0.5 mg/cm^2^ Pt-loaded carbon cloth [33] as the anode to avoid the restriction of the anode reaction, which is often observed in MFCs with low COD accessibility (Supplementary Figure S1). After operation for 35, 583, and 768 days, the cathodes and AEMs were set with the anode. The cathode and anode were used as the working electrode (WE) and counter electrode, respectively, and an Ag/AgCl reference electrode (012167RE-1B; BAS Co., Ltd., Tokyo, Japan) was placed near the anode. LSV measurements were performed using an electrochemical workstation (VMP-3; Bio-Logic, Claix, France) supplemented with hydrogen in the anolyte at approximately 0.1 MPa. The WE potential was set to 0.5 V and swept to −0.5 V vs. Ag/AgCl at 0.5 mV/s.

### 2.7. Investigation of Dirt on Membrane

White precipitates on 25 cm^2^ of the AEM (583 d) or CEM (35 d) were scrubbed and dissolved in 10 mL of 0.1 N HCl. The extracts were then filtered using a PTFE membrane filter (0.45 μm pore size) (Merck Millipore, Darmstadt, Germany). The filtrates were diluted 5–20 times and analyzed using an ion chromatography system (Shimadzu, Kyoto, Japan), including an electron conductivity detector (CDD-10Avp). Anions and cations in the samples were separated at 40 °C using Shim-pack IC-A3 (Φ4.6 × 150 mm) and Shim-pack IC-C4 (Φ4.6 × 150 mm), respectively. The mobile phase was a mixture of 8 mM p-hydroxybenzoic acid, 3.2 mM Bis-Tris, and 50 mM boric acid for anion analysis and a mixture of 2.5 mM oxalic acid dihydrate and 5 mM 18-Crown-6 for cation analysis.

The anode side of the AEM and CEM were observed by fluorescence microscopy after staining with SYBR Green II, a DNA-binding dye, as described in a previous study [34].

## 3. Results and Discussion

### 3.1. Current Production by the Two MFCs throughout the Operation

**Figure 1:**
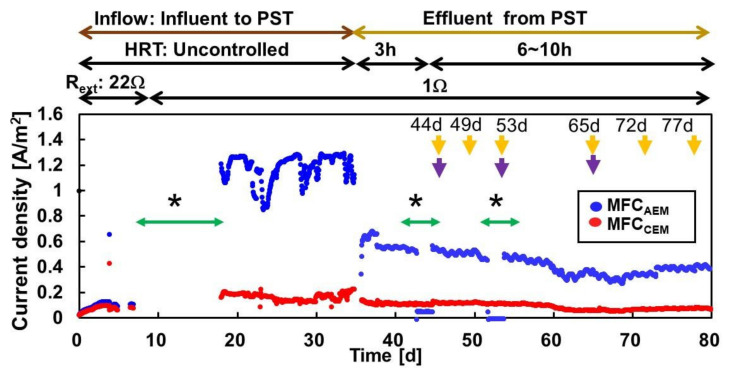
Current production by MFC_AEM_ and MFC_CEM_ throughout the operation. * Indicates the times when the data logger had trouble with the MFC. Purple and yellow arrows indicate the timing for PI curve or COD analysis, respectively.

The MFC_AEM_ and MFC_CEM_ produced 0.80–1.30 A/m^2^ of current with the PST influent, although the operation was terminated due to the frequent clogging in the influent tube. The MFC_AEM_ produced a current of 0.30–0.70 A/m^2^ with a continuous inflow of the PST effluent for 2.6–9.8 h of HRT. This current was approximately 4–5 times higher than that of MFC_CEM_, which produced 0.10–0.20 A/m^2^ (Figure 1). After 44 d, both MFC_AEM_ and MFC_CEM_ exhibited relatively stable currents, except for decreased currents in the MFC_AEM_ reactor due to pumping problems. The slightly lower current observed from days 60 to 70 was probably caused by the supplementation of low COD because of the dilution of the inflow by rainwater. Assuming similar anode resistances in the MFC_AEM_ and MFC_CEM_, the difference in current production can be attributed to the cathode reaction, which is typically regulated by the oxygen reduction rate of the cathode catalyst, oxygen availability, and ion mobility. The membrane resistance determined by ion mobility was smaller in CEM (0.16Ω·m^2^) than in AEM (0.26 Ω·m^2^) according to the manufacturer’s catalogue. Both MFCs had identical loaded cathode catalyst and oxygen availability. However, they had different binder components, that is, a mixture of PDDMAC and PTFE for MFC_AEM_, and Nafion for MFC_CEM_, which could potentially cause differences in oxygen availability to the carbon catalyst. Another factor was the difference in pH at the cathode. An approximate pH of 10–11 was measured on the cathode surface with CEM, in contrast to a neutral pH on that with AEM. This pH imbalance restricts electron transfer to oxygen, as suggested in a previous study [25]. Collectively, the differences in ion mobility, binder components, and pH resulted in higher current production in the MFC_AEM_.

The current density was similar to that generated by a similar MFC core without carbon brushes. The MFC without carbon brushes was also operated in the PST effluent with continuous inflow for 6 h of HRT using 2 Ω of the external resistance and recorded an average current density of 0.32 A/m^2^ [26]. The MFC_AEM_ used in this study produced an average of 0.38 A/m^2^ for 6 h of HRT. This was unexpected because the increase in the anodic area caused by introducing the carbon brushes has been demonstrated in several studies [35,36]. The similarity in current production, despite the increase in the anodic area, was due to the reaction restriction of the cathode.

### 3.2. COD Removal and CE

The COD_IN_ for the two MFCs was 210 ± 40 mg/L. MFC treatment at HRT = 7 h resulted in 66 ± 9.0 mg/L and 99 ± 51 mg/L of COD_EF_ for the MFC_AEM_ and MFC_CEM_, respectively (Table 1). The COD-REs of MFC_AEM_ and MFC_CEM_ were 69% ± 2.0% and 54% ± 19%, respectively, and were not significantly different (*p* > 0.05). The CEs of MFC_AEM_ and MFC_CEM_ were 1.6 ± 0.40% and 0.50 ± 0.35%, respectively. The superiority of MFC_AEM_ in current recovery indicates the higher CE in MFC_AEM_ than in MFC_CEM_.

The COD-RE achieved in the MFC indicated an improvement compared to that obtained with our previous MFC_AEM_, which exhibited 30% COD-RE at HRT = 6 h [26]; the COD-RE increased by approximately twice upon the introduction of carbon brushes. In addition, the CE was drastically reduced to less than 10% of that of the previous MFC without carbon brushes (23%) due to the reduction in specific cathode or separator areas with respect to wastewater volume; the introduction of 12 carbon brushes reduced the specific area from 14 m^2^/m^3^ [26] to 3.2 m^2^/m^3^ in this study. These results indicate the importance of determining the optimum ratio of carbon brushes to a specific cathode area [37]. However, the optimum ratio of carbon brushes to the MFC_AEM_ is expected to vary at different CODs in the MFC reactor. This necessitates comprehensive MFC performance modeling that integrates COD as well as the anode and cathode surface areas.

### 3.3. Polarization Curve

**Figure 2:**
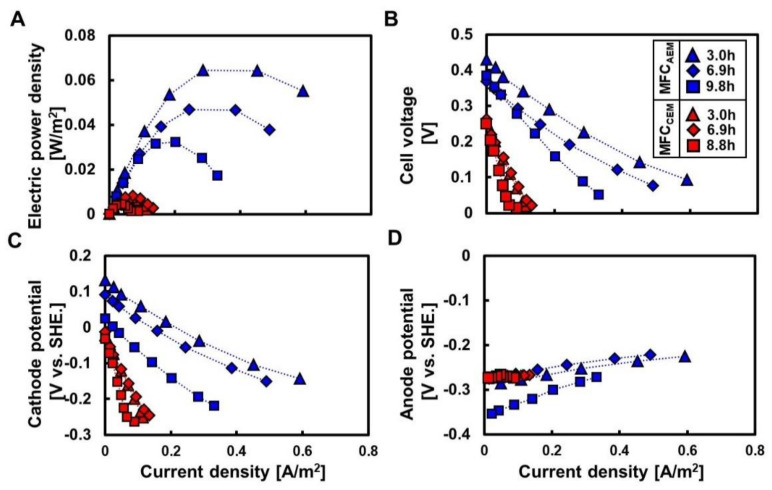
Effects of ion selectivity of the membrane separator on electricity and potential. Panels (**A**,**B**) present power density (**A**) and cell voltage (**B**) with varied currents, respectively. Panels (**C**,**D**) present cathode (**C**) and anode potential (**D**) at different current densities, respectively.

Figure 2 shows the polarization curves for the MFC_AEM_ and MFC_CEM_ at HRTs in the range of 3.0–9.8 h. The open-circuit voltage (OCV) of the MFC_AEM_ was the highest (0.43 V) for an HRT of 3.3 h and decreased to 0.37 and 0.39 V for an HRT of 6.9 and 9.8 h (Figure 2B, Table 1), respectively. The maximum power density (P_max_) showed a similar trend and was 0.064 W/m^2^ for an HRT of 3.3 h and decreased to 0.047 W/m^2^ and 0.032 W/m^2^ at 6.9 h and 9.8 h, respectively. The maximum current density (I_max_) decreased marginally with the increase in HRT; it was 0.59 A/m^2^ at 3.0 h and decreased to 0.49 A/m^2^ and 0.33 A/m^2^ at 6.9 h and 9.8 h, respectively. The HRT-dependent decrease in electricity generation has been repeatedly observed in the MFC in previous studies [2,22,26]. This decrease was revealed by the low COD due to the extended time required for microbial degradation.

The MFC_CEM_ had an OCV of 0.25–0.26 V, I_max_ of 0.089–0.13 A/m^2^, and P_max_ of 0.0037–0.0081 W/m^2^, regardless of the HRT (Figure 2A,B, Table 1). The OCV, I_max_, and P_max_ for the MFC_CEM_ were 60–70%, 22–26%, and 12–16% those of MFC_AEM_ at similar HRTs, respectively. The stable but lower OCV, I_max_, and P_max_ in the MFC_CEM_ indicates a limitation of the cathode reaction rate in the MFC_CEM_ because of the pH imbalance in the MFC_CEM_ [25].

The differences in the resistance between the MFC_AEM_ and MFC_CEM_ were considered by dividing the resistances by the anode (R_an_) and cathode (R_ca_) resistances. The R_an_ values of the MFC_AEM_ were 3.2, 3.4, and 6.9 mΩ·m^2^ at 3.3, 6.9, and 9.8 h, respectively. These values indicated that the COD accessibility in the anolyte affected the R_an_ (Figure 2D, Table 2). The R_an_ of the MFC_CEM_ was 1.7–1.9 mΩ∙m^2^ and lower than that of the MFC_AEM_. In contrast, the R_ca_ of MFC_AEM_ was 13–19 mΩ∙m^2^ and approximately 27–43% that of the MFC_CEM_ (41–49 mΩ·m^2^). These results indicated that the higher electricity production in the MFC_AEM_ can be attributed to the lower R_ca_ in the MFC_AEM_ (Figure 2C, Table 2).

The I_max_ and P_max_ of the MFC_AEM_ were in the ranges observed in our previous study without carbon brushes and with 91 mg/L of COD [22], that is, 0.19–0.38 A/m^2^ and 0.038–0.081 W/m^2^, respectively. The enhancement of the carbon brushes was restricted due to the low COD and limitation of the cathode reaction in the MFC_AEM_.

According to the results presented in Section 3.1, Section 3.2 and Section 3.3, the superiority of the AEM as a separator in an air-cathode MFC was apparent. Therefore, the resistance of the cathode in MFC_AEM_ was further investigated.

### 3.4. Linear Sweep Voltammetry

**Figure 3:**
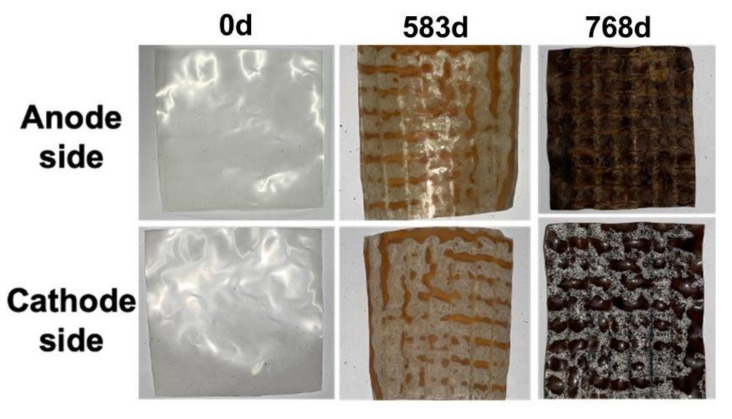
Change in AEM appearance with operation age.

The color change of the AEM from clear to dark brown (Figure 3) after extended operation of the MFC_AEM_ [22], motivated us to evaluate the effect of operation time on current production by the MFC_AEM_. The surface apparatus differed on either side of the AEM; the anode side was covered with a dark brown film, whereas the cathode side was covered with white precipitates. The effect of dirt on the current production was evaluated by LSV.

**Figure 4:**
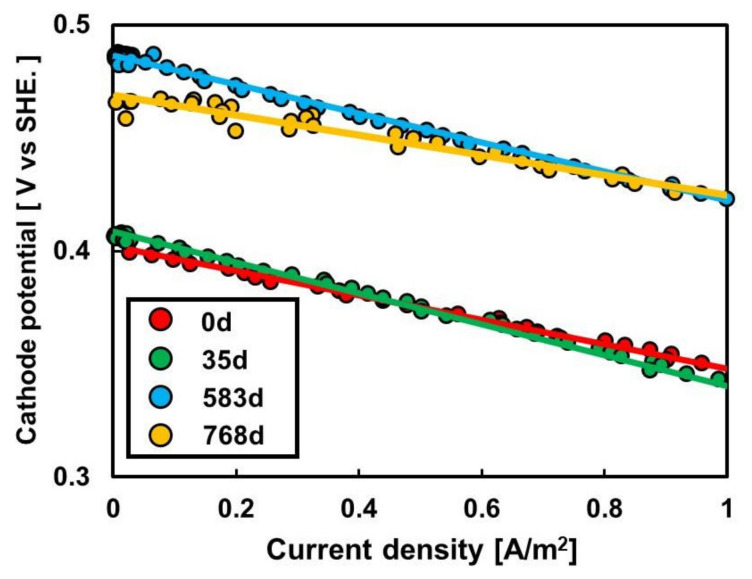
Effect of AEM age on cathode reaction in an H_2_-oxidizing air-cathode fuel cell filled with sewage wastewater as anolyte.

The LSV was performed using a small air-cathode fuel cell filled with sewage wastewater and using Pt as the anode catalyst under H_2_ supplementation to prevent the restriction of the microbial anode reaction (Supplementary Figure S1). The original AEM (AEM_0_) and AEMs (AEM_35_, AEM_583_, and AEM_768_) taken from the MFC_AEM_ after 35, 583, and 768 days of operation exhibited a marginal increase in the slope, i.e., the cathode resistance (Rca_-H2_), with the increase in operation time (Figure 4). The Rca_-H2_ for all AEMs remained apparently unchanged at 8.0 ± 2.0 mΩ·m^2^ (Figure 4, Table 2). A similar Rca_-H2_ indicates a minor effect of dirt on the cathode resistance. The potential of the cells with AEM_583_ and AEM_768_ was marginally higher than that of AEM_0_ and AEM_35_, although the mechanism behind it is unknown.

### 3.5. Membrane Resistance in NaCl Solution

**Figure 5:**
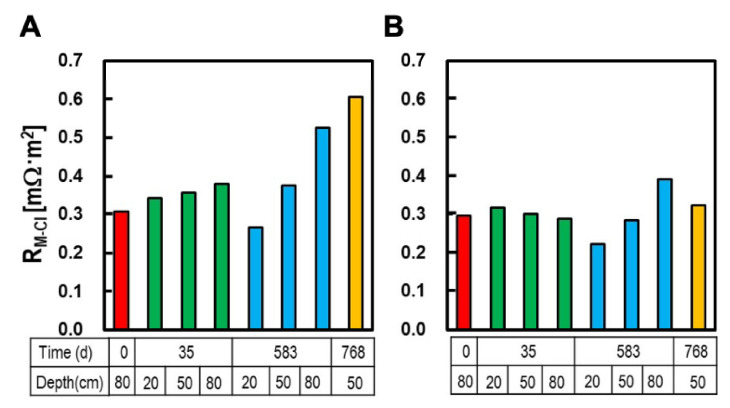
Membrane resistance (R_M-Cl_) as Cl^−^ mobility of AEMs taken from different depths and operation times. Panels (**A**,**B**) indicate R_M-Cl_ of the AEMs after 1 h and 6 weeks of immersion in 0.5 M NaCl, respectively.

The AEM resistance measured in terms of the Cl^−^ mobility in the 0.5 M NaCl solution. In this experiment, AEMs were taken from the MFC_AEM_ after operation times of 35, 583, and 768 d at depths of 20, 50, and 80 cm (Table 2 and Figure 5).

The membrane resistance (R_M-Cl_) of the original AEM was 0.31 mΩ·m^2^, which increased to 0.36, 0.37, and 0.61 mΩ·m^2^ in the MFC_AEM_ at a depth of 50 cm after 35, 583, and 768 d, respectively. R_M-Cl_ also tended to increase with increasing depth. For instance, for the MFC_AEM_ at 583 d, the R_M-Cl_ was 0.27 Ω·m^2^ at 20 cm, and it increased by 1.4 and 2.0 times at depths of 50 cm and 80 cm, respectively. This trend was also observed in the MFC_AEM_ at 35 d, but the depth-dependent rate of increase was less than that at 583 d. After six weeks of AEM immersion in 0.5 M of NaCl, the surface dirt peeled off from the AEM, which resulted in a 97–110% recovery of R_M-Cl_ compared to the original R_M-Cl_ of AEM_0_. This indicates that the surface dirt caused a significant increase in R_M-Cl_, in contrast to the marginal increase resulting from the irreversible damage caused to the membrane.

### 3.6. Investigation of Dirt on Membrane

Ion chromatograph analysis of white precipitates on the cathode side of the AEM and CEM revealed the presence of Ca^2+^, Mg^2+^, and Na^+^ as cations for both membranes (Supplementary Figure S2). The AEMs obtained from the MFC operated for 583 d exhibited 0.21, 0.11, and 0.0037 mg/cm^2^ of Ca^2+^, Mg^2+^, and Na^+^, respectively. It is hypothesized that the cations are transferred from wastewater and through the AEM by the extreme water pressure. The CEM at 35 d exhibited 0.29, 0.019, and 0.047 mg/cm^2^ for Ca^2+^, Mg^2+^, and Na^+^, respectively. Cl^−^ was detected as an anion because 0.1 M of HCl was used as the solvent for both membranes. In contrast, the anode side of the AEM and CEM exhibited an apparent increase in microbial density (Supplementary Figure S3). These results indicate that Ca, Mg, and Na salts that are eluted from wastewater appear as precipitates on the cathode side and that microbial biofilm appears as the brown dirt on the anode side.

## 4. Conclusions

The comparison of air-cathode MFC performances equipped with the AEM and CEM indicated the superiority of the AEM as a separator. The use of AEMs reduced the cathode resistance by eliminating the pH imbalance observed in an air-cathode MFC equipped with a CEM. Moreover, the AEM showed an increase in membrane resistance as the Cl^−^ mobility with the increase in operation time and depth. This result indicates a potential increase in membrane resistance when using the AEM in large-scale MFCs at the meter scale for extended periods. However, the increase in membrane resistance marginally contributes to the cathode resistance with OH^−^ movement for less than 768 d and at a depth of less than 1 m.

## Figures and Tables

**Table 1:** Summary of operation conditions of the MFC_AEM_ and MFC_CEM_ and the resulting performances in COD-RE, CE, OCV, I_max_, and P_max._

		Operation Time						
	IEM Type	44 d	53 d	65 d	49 d	72 d	77 d	Average (49, 72, 77 d)
HRT [h]	AEM	3.3	6.9	9.8	6.4	7.6	7.3	7.1 ± 0.7
	CEM	3.0	6.9	8.8	6.9	7.3	7.3	7.2 ± 0.3
COD_IN_ [mg/L]	AEM	-	-	-	230	230	170	210 ± 4 0
	CEM	-	-	-	230	230	170	210 ± 40
COD_EF_ [mg/L]	AEM	-	-	-	73	69	57	66 ± 9.0
	CEM	-	-	-	150	76	70	99 ± 51
COD-RE [%]	AEM	-	-	-	68	70	67	69 ± 2.0
	CEM	-	-	-	35	67	59	54 ± 19
CE [%]	AEM	-	-	-	1.7	1.2	1.8	1.6 ± 0.4
	CEM	-	-	-	0.85	0.27	0.46	0.50 ± 0.35
OCV [V]	AEM	0.43	0.37	0.39	-	-	-	-
	CEM	0.26	0.26	0.25	-	-	-	-
I_max_ [A/m^2^]	AEM	0.59	0.49	0.33	-	-	-	-
	CEM	0.13	0.11	0.089	-	-	-	-
P_max_ [W/m^2^]	AEM	0.064	0.047	0.032	-	-	-	-
	CEM	0.0081	0.0075	0.0037	-	-	-	-

**Table 2:** Summary of resistance measured in this study.

Time (d)	Depth [cm]	R_an_-MFC_CEM_ [mΩ·m^2^]	R_ca_-MFC_CEM_ [mΩ·m^2^]	R_an_-MFC_AEM_ [mΩ·m^2^]	R_ca_-MFC_AEM_ [mΩ·m^2^]	R_ca-H2_ [mΩ·m^2^]	R_M-Cl_ [mΩ·m^2^]	R_M-Cl_ * [mΩ·m^2^]
0						6.7	0.31	-
44		1.8	41	3.2	13		-	-
53		1.9	49	3.4	13		-	-
65		1.7	44	6.9	19		-	-
35	20						0.34	0.32
35	50					10	0.36	0.30
35	80						0.38	0.29
583	20						0.27	0.22
583	50					7.5	0.37	0.28
583	80						0.53	0.39
768	50					7.7	0.61	0.32

Depth: depth from the water surface [cm]; R_an_: anode resistance of the MFC; R_ca_: cathode resistance of the MFC; R_ca-H2_: cathode resistance of the H_2_ oxidizing fuel cell; R_M-Cl_: membrane resistance; * R_M-Cl_ measured after immersion for 6 weeks.

## Supplementary Materials

The following supporting information can be downloaded at: https://www.mdpi.com/article/10.3390/membranes12020183/s1, Figure S1: The illustration and apparatus of the reactor used for the LSV experiment; Figure S2: Identified ions precipitated on the cathode side of ion-exchange membranes in MFCs; Figure S3: Fluorescence microscopic images of microbes attached to the AEMs before MFC installation and 583 d after the operation.
