# Supplementary material for: Effect of Ion Selectivity on Current Production in Sewage Microbial Fuel Cell Separators
Source: Membranes (Basel). 2022 Feb 3;12(2):183. doi: 10.3390/membranes12020183 (PMC8878261; doi:10.3390/membranes12020183)
Supplement: Supplementary file 1 [file membranes-12-00183-s001.zip › membranes-1510886-supplementary.pdf]

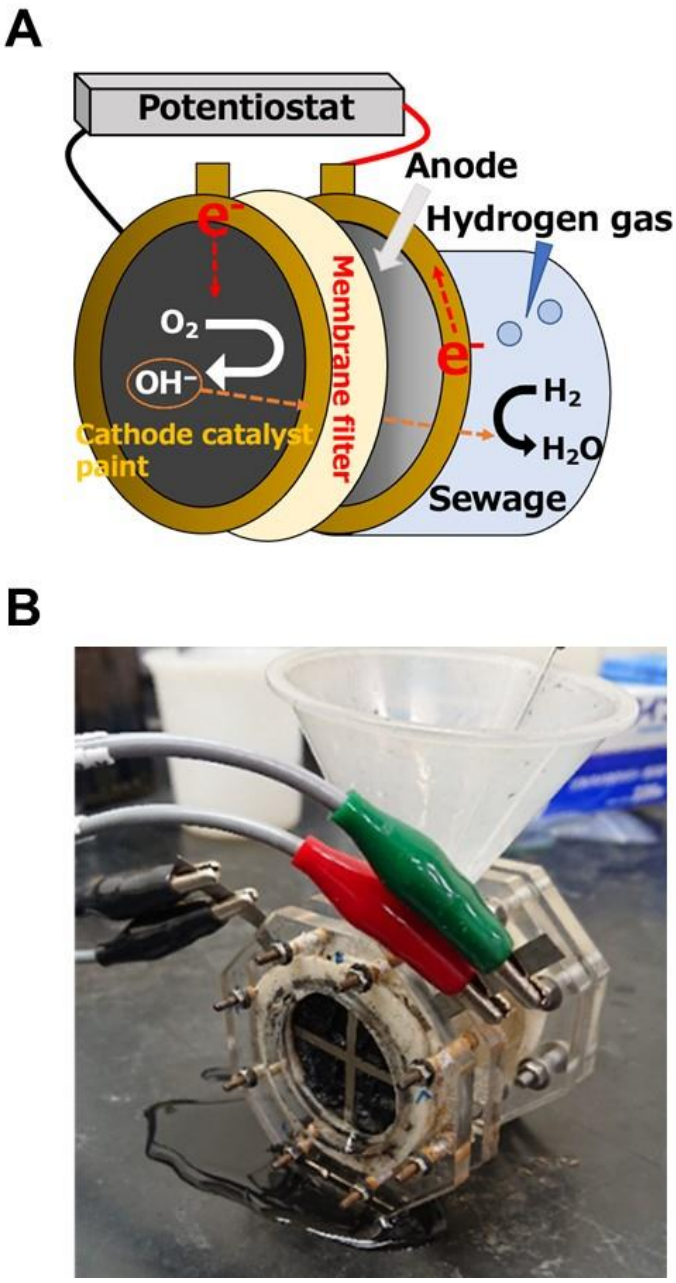

Supplement Figure S1: The illustration (A) and apparatus (B) of the reactor used for the LSV experiment.

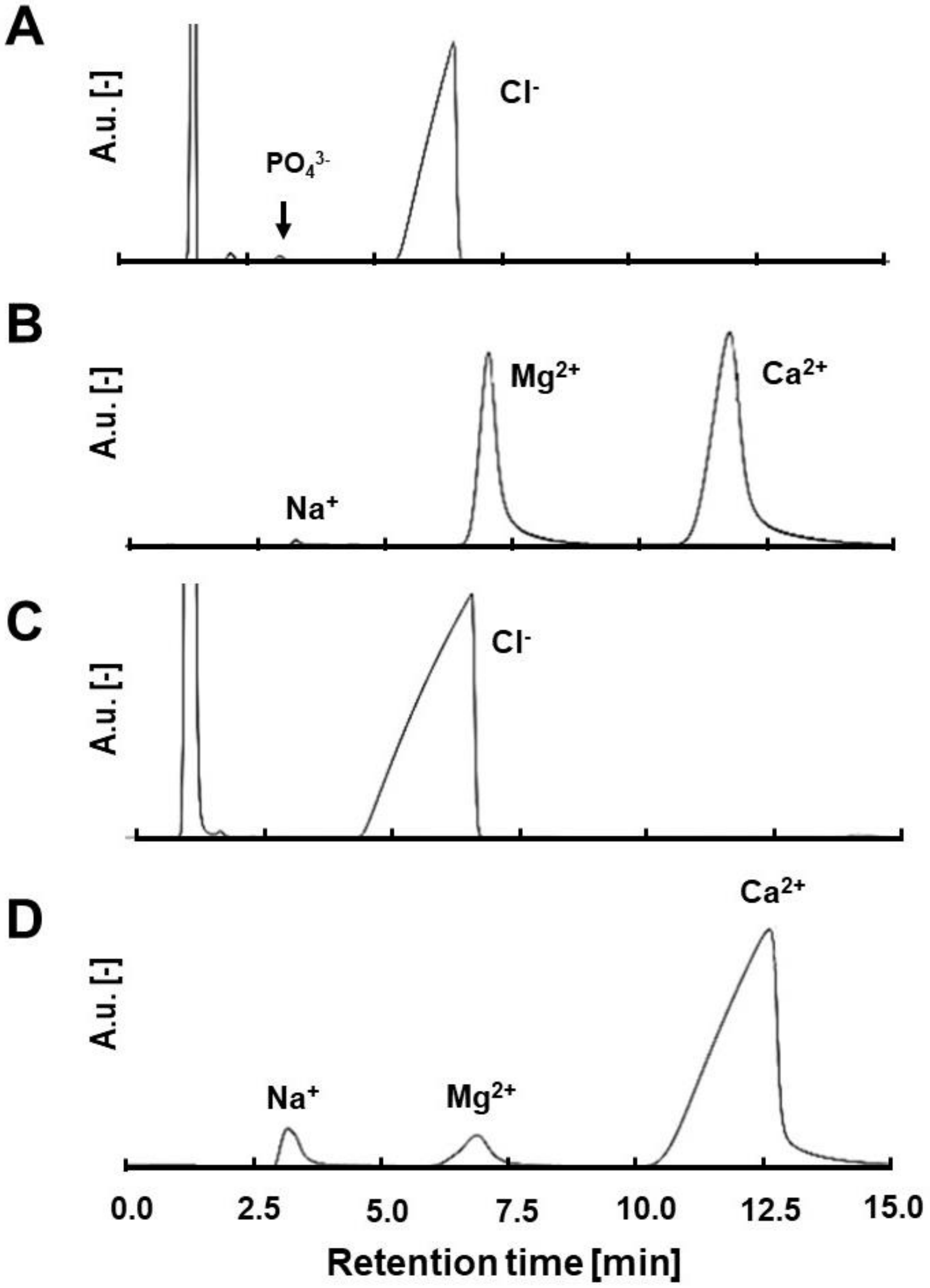

Supplement Figure S2: Identified ions precipitated on the cathode side of ion-exchange membranes in MFCs.

(A) Anions on AEM; (B) Cations on AEM; (C) Anions on CEM; (D) Cations on CEM

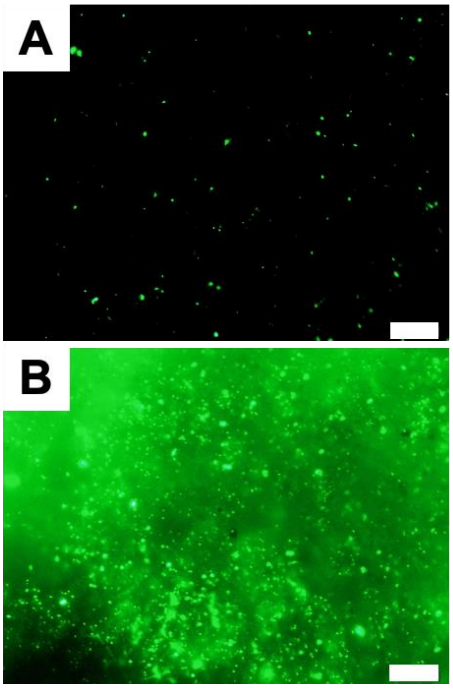

Supplement Figure S3: Fluorescence microscopic images of microbes attached to the AEMs before MFC installation (A) and 583d after the operation (B), respectively. The scale bar has 20 μm in length.
